# INTERNET USE AMONG BRAZILIANS ADULTS IN LATE LIFE: DETERMINANTS OF USE AND IMPACTS ON COGNITIVE HEALTH

**DOI:** 10.1093/geroni/igae098.1156

**Published:** 2024-12-31

**Authors:** Xiayu Chen, Mateo Farina, Flávia Andrade, Kun Wang

**Affiliations:** University of Illinois Urbana-Champaign, Champaign, Illinois, United States; University of Texas at Austin, Austin, Texas, United States; University of Illinois at Urbana Champaign, Urbana, Illinois, United States; Binghamton University, Binghamton, New York, United States

## Abstract

Growing numbers of older adults use the Internet daily, suggesting its potential to improve general cognitive functioning, memory, and verbal fluency. Yet, a significant gap exists in understanding the association between Internet use and cognitive health within low- and middle-income countries. This study addresses this gap and evaluates the association between Internet use and cognitive health among Brazilian older adults using data from the second wave of the Brazilian Longitudinal Study of Aging(ELSI-Brazil). Multivariate linear regressions were employed to evaluate the association of Internet use with cognitive function, memory, and verbal fluency. Blinder-Oaxaca decomposition was conducted to estimate and decompose the cognitive health disparity between Internet users and non-users. Our finding suggested that about 17% of the older adults in the sample were Internet users, and 69% of them use the Internet on a daily basis. Internet use was positively associated with general cognitive functioning (β=0.14, 95% CI 0.07-0.20) and memory (β =0.19, 95% CI 0.10-0.28), accounting for demographic, social, geographical, and health factors. The daily Internet use was positively associated with general cognitive function (β=0.14, 95% CI 0.06-0.22) and memory (β=0.19, 95% CI 0.09-0.29). However, no statistically significant association was found between Internet use, use frequency and verbal fluency. Our study has also found that educational and health attainment significantly explains the cognitive health disparity between Internet users and non-users among Brazilian older adults. The findings underscore the need for targeted strategies to bridge the digital divide and enhance cognitive health, particularly among socioeconomically and health-disadvantaged groups in Brazil.

